# Association Between Breastfeeding and Reduced Distal Sensory Polyneuropathy in Postmenopausal Women Aged 40–70 Years: Analysis of Data from the 1999–2004 National Health and Nutrition Examination Survey

**DOI:** 10.1089/bfm.2022.0228

**Published:** 2023-01-12

**Authors:** Jiayu Li, Somsook Santibenchakul, Yuda Chongpison, Jakkrit Amornvit, Sukanya Chaikittisilpa, Unnop Jaisamrarn

**Affiliations:** ^1^ Department of Obstetrics and Gynecology, Faculty of Medicine, Chulalongkorn University, Bangkok, Thailand.; ^2^ Department of Obstetrics and Gynecology, Faculty of Medicine, Chulalongkorn University, King Chulalongkorn Memorial Hospital, Bangkok, Thailand.; ^3^ Center of Excellence in Biostatistics, Research Affairs, Faculty of Medicine, Chulalongkorn University, Bangkok, Thailand.; ^4^ The Skin and Allergy Research Unit, Faculty of Medicine, Chulalongkorn University, Bangkok, Thailand.; ^5^ Division of Neurology, Department of Medicine, Faculty of Medicine, Chulalongkorn University, Bangkok, Thailand.; ^6^ King Chulalongkorn Memorial Hospital, Thai Red Cross Society, Bangkok, Thailand.; ^7^ Menopause Research Group, Department of Obstetrics and Gynecology, Faculty of Medicine, Chulalongkorn University, King Chulalongkorn Memorial Hospital, Bangkok, Thailand.

**Keywords:** breastfeeding, distal sensory polyneuropathy, peripheral neuropathy, National Health and Nutrition Examination Survey

## Abstract

**Background:**

Distal sensory polyneuropathy (DSP) is a common peripheral neuropathy subtype. We aimed to determine the association between breastfeeding and DSP among postmenopausal women aged 40–70 years, and the effect modification of obesity on this association.

**Methods:**

A cross-sectional study was conducted using data from the National Health and Nutrition Examination Survey 1999–2004. Postmenopausal women aged 40–70 years were included. Women with diabetes, stroke, cancer, cardiovascular disease, thyroid disease, liver disease, weak/failing kidneys, or amputation were excluded. Binary logistic regression was used to analyze the association between breastfeeding and DSP.

**Results:**

Among 798 participants, 386 (44.30%) reported breastfeeding history and 51 (5.29%) were defined as having DSP using the monofilament test. A significant inverse association was observed between breastfeeding and DSP (odds ratio [OR] = 0.29; 95% confidence interval [CI]: 0.11–0.79; *p* = 0.017) after adjusting for other confounding variables. In subgroup analysis, this adjusted association was observed only in the obese group (OR = 0.21; 95% CI: 0.06–0.73, *p* = 0.013).

**Conclusions:**

Breastfeeding was found to have potential benefits in the presence of DSP in postmenopausal women aged 40–70 years, and obesity modified the association between breastfeeding and DSP. Promoting breastfeeding may reduce the burden of peripheral neuropathy in middle-aged postmenopausal women.

## Introduction

Distal sensory polyneuropathy (DSP) is one of the most common subtypes of peripheral neuropathy characterized by symmetric distal foot or toe numbness, tingling, with/without neuropathic pain, or loss of sensation.^1^ Some patients may only experience mild paresthesia or negative symptoms; however, ignoring these signs may eventually put them at risk of disability or threaten their life.^2^ In 2010, ∼18.6 million U.S. adults were identified as having DSP by monofilament insensitivity.^3^

More than 10% of U.S. adults aged 40–69 years suffer from DSP, and this prevalence could reach to 30% among individuals aged ≥70 years.^3^ Diabetes is the most common cause for DSP and diabetic DSP is mostly irreversible.^4^ The prevalence of DSP increases among postmenopausal women, which may be related to estrogen deficiency.^5–7^ In addition, unhealthy lifestyle and lower socioeconomic status are also commonly associated with DSP.^8^

Approximately 11.8% of nondiabetic adults aged ≥40 years suffer from DSP.^3^ A growing body of literature links prediabetes and cryptogenic sensory polyneuropathy before the onset of frank diabetes.^9^ Obesity is another independent risk factor for nondiabetic DSP.^10^ Preventing underlying metabolic changes is an effective therapeutic strategy for peripheral neuropathy.^10^ Beneficial effects of breastfeeding on impaired insulin sensitivity and glucose tolerance have been found in nondiabetic postmenopausal women.^11,12^

Lactating animals have smaller adipocytes and lower peripheral lipoprotein lipase activity than nonlactating controls.^13^ Breastfeeding could enhance calorie expenditure, helps with weight loss, and changes body fat distribution.^14^ Differences in breastfeeding-related obesity have been observed in postmenopausal women.^11,14^ However, there are no reports in the literature on the association between breastfeeding and DSP among postmenopausal women.

Accordingly, this study aimed to investigate the association between breastfeeding history and DSP among postmenopausal women aged 40–70 years, using data from the National Health and Nutrition Examination Survey (NHANES) 1999–2004. Since breastfeeding is helpful in reducing the risk of obesity, a well-known risk factor for peripheral nerve injury,^11,15^ we also performed subgroup analyses to address the heterogeneity of the association between breastfeeding history and DSP in obese and nonobese subgroups.

## Materials and Methods

### Participants

**FIG. 1.**
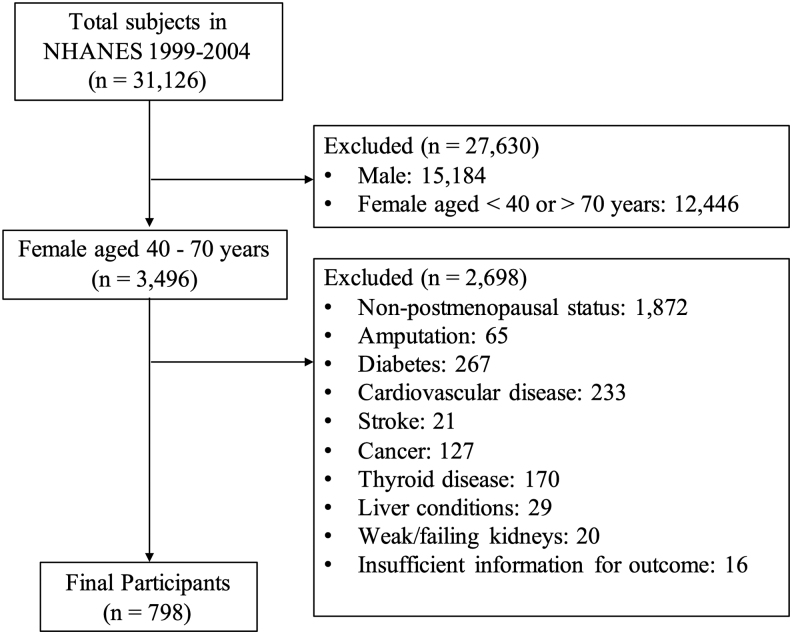
Flow diagram for eligible participants.

Data from the NHANES 1999–2004 were used in this cross-sectional study.^16^ We included postmenopausal women aged 40–70 years. Women with stroke, cancer, diabetes, cardiovascular disease, thyroid disease, liver disease, or weak/failing kidneys were excluded because these diseases are potential causes of peripheral neuropathy.^2^ Women with amputation or insufficient information regarding their eligibility and outcomes were excluded. All data regarding the mentioned health conditions were collected as self-reported medical histories. Ultimately, 798 participants were included in this study (Fig. 1). The final analytic sample size was without missing values in the outcome, but with missing values in some other variables (Supplementary Table S1).

The NHANES study protocol was approved by the institutional review board of the Centers for Disease Control and Prevention, and all the participants provided informed consent. This study was exempted by the institutional review board of the faculty of medicine, Chulalongkorn University (No. 0422/65). This study was reported following the reporting guidelines of RECORD statement.^17^

### Outcome

DSP was defined as having at least one insensate site on either foot, as determined by a 10-g monofilament.^3^ The insensate site was determined when the participants were unable to respond correctly to filament pressure at three plantar sites (the first metatarsal head, fifth metatarsal head, and hallux). Details of the outcome assessment can be found in a previous study.^18^

### Exposure

Breastfeeding history was defined as having ever breastfed any of their children (never and ever).

### Covariates

Age was defined in years at the time of household interview (40–59 and 60–70 years).^3^ Race was derived according to race and Hispanic origin (Hispanic, including Mexican American and other Hispanic; non-Hispanic, including the non-Hispanic white, non-Hispanic black, and other races). Education was defined as highest degree received (less than high school and high school or higher). Income was defined as prescribed investor rate (PIR), a ratio of family income to the poverty threshold (PIR ≤2.00 and PIR >2.00, sample median cutoff values). Insurance was defined as coverage by health insurance or some other kind of health care plan (covered and not covered).

Alcohol use was defined as consumption of at least 12 drinks of any type of alcohol (including liquor, beer, wine, wine coolers, and any other type of alcoholic beverage) throughout life. A 355.2 ml of beer, 118.4 ml of wine, or 29.6 ml of liquor is seen as a drink (never and ever). Smoking was defined as having smoked at least 100 cigarettes throughout life or smoking at the time of interview (never or ever). Hypertension was defined as an average systolic blood pressure ≥140 mmHg, an average diastolic blood pressure ≥90 mmHg, or self-reported use of prescribed medication for high blood pressure (yes or no).

Body mass index (BMI) was calculated as weight in kilograms divided by height in meters squared (nonobese: <25 kg/m^2^; obese: ≥25 kg/m^2^).^19^ Gravidity was defined as sum of the number of pregnancies, including current pregnancy, live birth, miscarriage, stillbirth, tubal pregnancy, and abortion (<4 or ≥4).^20^ Time since menopause in years was calculated as age at interview minus age at last menstrual period (≤20 and >20 years).^21^ A history of exogenous hormone use was defined as use of contraceptives, including birth control pills and contraceptive injection, or any type of menopause hormone therapy, including pills, cream, patches, and injectables (never or ever).

### Statistical analysis

The standard software package (Stata/SE 15.1 for Mac; StataCorp) was used for data analysis. All analyses were appropriately adjusted for the survey weights, clusters, and strata, as the NHANES employed a complex multistage probability sampling design to recruit the participants. In a complex survey, the precision of the estimated variance is related to degrees of freedom (*df*), which depends on information about the first stage of sample design (*df = #primary sampling units - #strata*). If an estimated standard error has less than eight degrees of freedom, the computed estimate may be unreliable.^22^

All categorical data were summarized as weighted proportions with 95% confidence intervals (CI).^23^ The weighted median with interquartile range was reported for the numerical data. Using the Hosmer's strategy, design-based binary logistic regression was conducted to test the association between breastfeeding history and DSP.^24,25^ We selected potential confounders in the univariate analysis using the purposeful selection of covariates strategy (*p* < 0.25). Possible interactions were tested to identify whether the effect of each variable was not constant over BMI levels, based on both clinical and statistical considerations.

A significant interaction term between BMI and time since menopause was identified. We conducted a subgroup analysis to investigate whether obese status modifies the association between breastfeeding history and DSP. We used a similar model (except for BMI) for subgroup analysis. The goodness of fit was tested for each model. The results are presented as odds ratios (ORs) with 95% CI. Statistical significance was set at a two-sided *p*-value of <0.05.

## Results

In total, 798 participants were included in the analysis. There were 278 individuals in the NHANES 1999–2000, 259 in the NHANES 2002–2003, and 261 in the NHANES 2003–2004 cycles. The characteristics of the study population were summarized as frequencies with weighted prevalence given in Table 1. Of these, 386 (44.30%) participants reported a breastfeeding history, and 51 (5.29%) were defined as having DSP by the monofilament test.

**Table 1.** Characteristics of the Study Participants (NHANES 1999–2004 Cycle, *N* = 798)

Variables	*N*	%
Age (years)		
40–59	400	64.45
60–70	398	35.55
Race/ethnicity		
Hispanic	225	10.83
Non-Hispanic	573	89.17
Education		
Less than high school	260	20.40
High school and above	538	79.60
Income status		
PIR ≤2.00	268	24.61
PIR >2.00	451	65.79
Insurance		
Not covered	148	13.09
Covered	646	86.55
Alcohol use		
Never	187	19.54
Ever	610	80.11
Smoking		
Never	456	52.67
Ever	341	47.30
Hypertension		
No	380	54.96
Yes	402	42.92
BMI		
Nonobese	228	31.62
Obese	559	67.06
Gravidity		
<4	438	64.32
≥4	360	35.68
Breastfeeding history		
Never	332	43.24
Ever	386	44.30
Time since menopause (years)		
≤20	626	82.47
>20	144	14.22
History of exogenous hormone use		
Never	150	13.43
Ever	647	86.27
DSP		
Non-DSP	747	94.71
DSP	51	5.29

Number (*N*) of participants with weighted percentages (%). Column percentages for the sample totals that do not add up to 100% are a result of missing data.

DSP, distal sensory polyneuropathy; PIR, prescribed investor rate; BMI, body mass index.

The weighted median (interquartile range) of age and time since menopause was 56 (51–62) and 8 (3–16) years, respectively. The prevalence of DSP was significantly higher among Hispanic race and those without breastfeeding history (*p* < 0.05) (Table 2). Women of Hispanic race and gravidity ≥4 were more likely to breastfeed their children (*p* < 0.05) (Supplementary Table S2).

**Table 2.** Characteristics of Study Participants by Distal Sensory Polyneuropathy (NHANES 1999–2004 Cycle, *N* = 798)

Variables	Non-DSP,* N* = 747		DSP,* N* = 51		*p*-value
	%	95% CI	%	95% CI	
Age (years)					0.490
40–59	95.24	91.85–97.51	4.76	2.49–8.15	
60–70	93.76	90.18–96.33	6.24	3.67–9.82	
Race/ethnicity					0.045^*^
Hispanic	89.91	80.52–95.76	10.09	4.24–19.48	
Non-Hispanic	95.30	93.17–96.92	4.70	3.08–6.83	
Education					0.265
Less than high school	92.50	85.79–96.69	7.50	3.31–14.21	
High school and above	95.28	92.86–97.07	4.72	2.93–7.14	
Income status					0.301
PIR ≤2.00	93.17	86.91–97.03	6.83	2.97–13.09	
PIR >2.00	95.66	92.90–97.58	4.34	2.42–7.10	
Insurance					0.994
Not covered	94.68	87.19–98.47	5.32	1.53–12.81	
Covered	94.70	92.61–96.33	5.30	3.67–7.39	
Alcohol use					0.887
Never	94.37	87.84–98.01	5.63	1.99–12.16	
Ever	94.77	91.88–96.87	5.23	3.13–8.12	
Smoking					0.152^*^
Never	95.93	93.62–97.59	4.07	2.41–6.38	
Ever	93.35	89.13–96.31	6.65	3.69–10.87	
Hypertension					0.402
No	93.77	89.94–96.46	6.23	3.54–10.06	
Yes	95.69	92.10–97.96	4.31	2.04–7.90	
BMI					0.378
Nonobese	96.38	91.99–98.74	3.62	1.26–8.01	
Obese	94.19	90.96–96.53	5.81	3.47–9.04	
Breastfeeding history					0.034^*^
Never	93.23	89.41–96.00	6.77	4.00–10.59	
Ever	96.92	94.06–98.65	3.08	1.35–5.94	
Gravidity					0.989
<4	94.69	90.41–97.45	5.31	2.55–9.59	
≥4	94.72	91.74–96.87	5.28	3.13–8.26	
Time since menopause (years)					0.095^*^
≤20	95.35	93.33–96.91	4.65	3.09–6.67	
>20	91.71	84.84–96.14	8.29	3.86–15.16	
History of exogenous hormone use					0.306
Never	95.35	93.33–96.91	4.65	3.09–6.67	
Ever	95.03	92.62–96.84	4.97	3.16–7.38	

Values are weighted row percentages with 95% CI.

^*^ *p* < 0.25 versus the values of the non-DSP group.

CI, confidence intervals.

In the final model, 613 participants from 43 strata were included. A postmenopausal woman aged 40–70 years who had breastfed any of her child experienced a 71% reduction in the odds of having DSP compared with those without any breastfeeding history (adjusted OR = 0.29; 95% CI: 0.11–0.79, *p* = 0.017), after adjusting for age, income, race, BMI, and time since menopause, in the primary analysis multivariable model (Table 3).

**Table 3.** Associations Between Breastfeeding and Distal Sensory Neuropathy (NHANES 1999–2004 Cycle)

Analysis strategies	Crude OR (95% CI)	*p*-value	Adjusted OR (95% CI)	*p*-value
Primary analysis				
Breastfeeding (Ever vs. never)^a^	0.44 (0.20–0.95)	0.038^*^	0.29 (0.11–0.79)	0.017^*^
Effect modification by BMI^b^				
Breastfeeding (Ever vs. never) within nonobese group	1.12 (0.23–5.42)	0.885	0.55 (0.09–3.43)	0.513
Breastfeeding (Ever vs. never)^c^ within obese group	0.36 (0.14–0.93)	0.035^*^	0.21 (0.06–0.73)	0.013^*^

Values are weighted OR with 95% CI.

^a^ Adjusted for age, income, race, BMI, and time since menopause in the primary multivariable model analysis, *n* = 613.

^b^ Adjusted for age, income, race, and time since menopause in the subgroup analysis multivariable model; *n* = 169 in the nonobese group and *n* = 444 in the obese group.

^c^ Among the 43 strata in the primary analysis, 3 strata were omitted in the nonobese group analysis multivariable model because they contained no subpopulation members. ^*^p < 0.05 versus the values of nondistal sensory polyneuropathy group.

OR, odds ratio.

In the subgroup analysis, lower odds of DSP among women with breastfeeding history were seen in both subgroups; however, a significant adjusted association between breastfeeding and DSP was only observed in the obese group (adjusted OR = 0.21; 95% CI: 0.06–0.73, *p* = 0.013), with a total of 444 participants from 43 strata. In the multivariable analysis of the nonobese group, the adjusted association was not statistically significant (adjusted OR = 0.55; 95% CI: 0.09–3.43, *p* = 0.513), with a total of 169 participants from 40 strata.

## Discussion

In this cross-sectional study, we found that postmenopausal women aged 40–70 years who had breastfed any of their children experienced a reduced risk of DSP compared with those without a breastfeeding history. In the subgroup analysis, this potential beneficial effect of breastfeeding on DSP was found only in the obese group.

The underlying mechanism of the association between breastfeeding and DSP among nondiabetic postmenopausal women remains unclear. Prediabetes and metabolic syndrome components (besides diabetes) have been reported to be the major causes of nondiabetic cryptogenic sensory polyneuropathy.^10^ In a normoglycemic animal model, self-reinforcing cascade of metabolic and inflammatory effects ultimately resulted in microvascular damage and peripheral nerve dysfunction.^10^ The effects of breastfeeding on regulation of maternal metabolism may explain our findings.^26,27^

Animal models suggested that blood glucose and insulin levels were reduced by 20% and 35% in lactating rats, respectively.^28^ Breastfeeding has been inversely associated with impaired insulin sensitivity and glucose tolerance in postmenopausal women.^11,12^ Breastfeeding has a beneficial effect on hyperlipidemia, visceral fat, and central obesity.^14^ In addition, among women with exclusive breastfeeding, a longer breastfeeding history might prevent peripheral nerve aging by slowing the return of ovulation after delivery and reproductive aging.^29^

Increased prolactin and oxytocin levels during breastfeeding may be involved in the peripheral neuroprotection of breastfeeding.^30–34^ Inflammation and oxidative stress play crucial roles in peripheral neuropathy.^10^ As a mediator of inflammation, tumor necrosis factor (TNF-α) can directly cause peripheral nerve injury and influence peripheral nerves by causing endothelial dysfunction and vascular thickening.^35^ Oxytocin acts as a neuroprotective factor by preventing ischemia-induced inflammation and oxidative stress by regulating NF-κB, TNF-α, IL-1β, and microglial activation.^30–32^

Oxytocin might prevent inflammation in the peripheral nervous system by reducing the production of norepinephrine, which has been positively associated with the aggravation of symptoms in neuritis.^31,36^ In addition, prolactin provides strong neuronal protection as a neuropeptide with antiapoptotic, anti-inflammatory, and antioxidant properties.^33,34^

It is challenging to reverse established neuropathy; however, the preventive factor might play a role in preventing the occurrence of DSP and downregulating its development.^10^ Hence, we hypothesized that breastfeeding would reverse metabolic disorders and exert anti-inflammatory effects during the premenopausal phase. Conversely, adverse changes persist for longer periods, and the risk of developing DSP increases when women do not breastfeed.^14^ Postmenopausal women aged 40–70 years were also included in our study. Age >70 years is a well-known risk factor for DSP; hence, stronger risk factors that counteract the beneficial effects of breastfeeding on DSP may be present in women over the age of 70 years.^3^

Subgroup analysis results showed that obesity status based on BMI seemed to modify the effect of breastfeeding on DSP. Specifically, in our study, lower odds of DSP among breastfeeding women were observed in the obese group (BMI ≥25 kg/m^2^). In the nonobese group, the association between breastfeeding and DSP was not significant in postmenopausal women aged 40–70 years. Overweight or obese women suffer from more metabolic disorders and inflammation than underweight and normal weight women.^10,37^ Therefore, the results of the subgroup analysis might support that lactation exerts a stronger protective effect on peripheral nerves among those with more metabolic and inflammatory issues. The protection of breastfeeding may work by regulating metabolism and inflammation.

This study has several strengths. First, all the estimates in our study are nationally representative. Second, we used logistic regression with a purposeful selection strategy to assess the association between exposure and outcome. This strategy allowed us to correctly identify and retain confounders at a higher rate than other selection algorithms. Lastly, we identified that the effect of breastfeeding was not constant over BMI levels by subgroup analysis.

Our study had several limitations. First, the assessment of peripheral neuropathy was limited to the monofilament test and was only available in the 1999–2004 NHANES. Other neurological examinations, nerve conduction, or biopsies were not available from the NHANES.^38^ Second, information and recall bias in assessing exposure existed among postmenopausal women, and breastfeeding duration or breastfeeding patterns were not specified in this study. Third, as the sample size of participants included in the fasting subsample was small, study participant characteristics based on blood tests were not provided in this study.

Our study population did not undergo fasting blood tests or insulin sensitivity tests. Diabetes was defined by history obtained through interviews. Therefore, nondiabetic status may be misclassified. Our study did not include blood cholesterol or other inflammatory biochemical markers as potential confounders, which may explain the underlying mechanisms of DSP. Malnutrition is another common cause of DSP^2^; however, participants with vitamin B_12_ deficiency were not specified or excluded from our study. Fourth, as this was a cross-sectional study, only the prevalence of DSP was reported.

The causal relationship could not be confirmed; therefore, a prospective cohort study is required. Fifth, the study population included only postmenopausal women in the United States, and breastfeeding rates varied by race and ethnicity among U.S. women.^39^ Therefore, future studies should be conducted among different ethnic groups and racial differences should be considered. Sixth, all the participants were collapsed into two category levels for each variable because the sample size was small with more specific subdomains.^40^ Future studies should consider participants' characteristics in specific categories of each variable. Lastly, we cannot rule out the possibility that the association between breastfeeding and DSP in the subgroup analysis was random, despite this difference being related to metabolism.

## Conclusion

In conclusion, breastfeeding was found to have potential benefits in the presence of DSP in postmenopausal women aged 40–70 years, and obesity modified the association between breastfeeding and DSP. The promotion of breastfeeding may reduce the burden of peripheral neuropathy in middle-aged postmenopausal women. Further studies with definite DSP diagnoses and more details of breastfeeding duration or patterns should be conducted to confirm this association.

## Supplementary Material

Supplemental data

Supplemental data
