# Supplementary material for: Association Between Breastfeeding and Reduced Distal Sensory Polyneuropathy in Postmenopausal Women Aged 40–70 Years: Analysis of Data from the 1999–2004 National Health and Nutrition Examination Survey
Source: Breastfeed Med. 2023 Jan 12;18(1):59–65. doi: 10.1089/bfm.2022.0228 (PMC9889012; doi:10.1089/bfm.2022.0228)
Supplement: Supplemental data [file Supp_TableS1.docx]

**Supplemental Table 1. Missingness of all variables (NHANES 1999–2004 cycle, N= 798)**

| **Variables** | **N** | **%** |
| --- | --- | --- |
| Age | 0 | 0.00 |
| Race/Ethnicity | 0 | 0.00 |
| Education | 0 | 0.00 |
| Income | 79 | 9.90 |
| Insurance | 4 | 0.50 |
| Alcohol | 1 | 0.13 |
| Smoking | 1 | 0.13 |
| Hypertension | 16 | 2.01 |
| BMI | 11 | 1.38 |
| Gravidity | 0 | 0.00 |
| Breastfeeding | 80 | 10.03 |
| Time since menopause | 28 | 3.51 |
| History of exogenous hormone use | 1 | 0.13 |
| DSP | 0 | 0.00 |

Values are presented as number (n) and percentage (%). DSP, distal sensory polyneuropathy; BMI, body mass index.
