# Supplementary material for: Association Between Breastfeeding and Reduced Distal Sensory Polyneuropathy in Postmenopausal Women Aged 40–70 Years: Analysis of Data from the 1999–2004 National Health and Nutrition Examination Survey
Source: Breastfeed Med. 2023 Jan 12;18(1):59–65. doi: 10.1089/bfm.2022.0228 (PMC9889012; doi:10.1089/bfm.2022.0228)
Supplement: Supplemental data [file Supp_TableS2.docx]

**Supplemental Table 2. Characteristics of study participants by breastfeeding (NHANES 1999–2004 cycle, N=718)**

| **Variables** | **Never, N=332** | | **Ever, N=386** | | ***p*-value** |
| --- | --- | --- | --- | --- | --- |
|  | % | 95% CI | % | 95% CI |  |
| Age |  |  |  |  | 0.760 |
| 40-59 y | 48.78 | 41.32 - 56.29 | 51.22 | 43.71 - 58.68 |  |
| 60-70 y | 50.43 | 41.42 - 59.41 | 49.57 | 40.59 - 58.58 |  |
| Race/Ethnicity |  |  |  |  | 0.044* |
| Hispanic | 37.95 | 27.35 - 49.46 | 62.05 | 50.54 - 72.65 |  |
| Non-Hispanic | 50.89 | 44.42 - 57.35 | 49.11 | 42.65 - 55.58 |  |
| Education |  |  |  |  | 0.701 |
| Less than high school | 50.94 | 41.69 - 60.14 | 49.06 | 39.86 - 58.31 |  |
| High school and above | 48.96 | 42.06 - 55.89 | 51.04 | 44.11 - 57.94 |  |
| Income status |  |  |  |  | 0.248 |
| PIR ≤2.00 | 52.72 | 42.76 - 62.51 | 47.28 | 37.49 - 57.24 |  |
| PIR >2.00 | 47.35 | 40.51 - 54.27 | 52.65 | 45.73 - 59.49 |  |
| Insurance |  |  |  |  | 0.060 |
| Not covered | 57.85 | 46.30 - 68.80 | 42.15 | 31.20 - 53.70 |  |
| Covered | 47.98 | 41.68 - 54.33 | 52.02 | 45.67 - 58.32 |  |
| Alcohol use |  |  |  |  | 0.196 |
| Never | 43.68 | 32.95 - 54.87 | 56.32 | 45.13 - 67.05 |  |
| Ever | 50.71 | 44.29 - 57.11 | 49.29 | 42.89 - 55.71 |  |
| Smoking |  |  |  |  | 0.103 |
| Never | 45.23 | 37.67 - 52.95 | 54.77 | 47.05 - 62.33 |  |
| Ever | 54.31 | 45.33 - 63.08 | 45.69 | 36.92 - 54.67 |  |
| Hypertension |  |  |  |  | 0.514 |
| No | 50.74 | 44.27 - 57.19 | 49.26 | 42.81 - 55.73 |  |
| Yes | 48.05 | 39.73 - 56.45 | 51.95 | 43.55 - 60.27 |  |
| BMI |  |  |  |  | 0.074 |
| Non-obese | 43.15 | 35.37 - 51.19 | 56.85 | 48.81 - 64.63 |  |
| Obese | 52.01 | 44.89 - 59.08 | 47.99 | 40.92 - 55.11 |  |
| Gravidity |  |  |  |  | 0.001* |
| <4 | 55.2 | 48.69 - 61.59 | 44.8 | 38.41 - 51.31 |  |
| ≥4 | 40.85 | 33.18 - 48.87 | 59.15 | 51.13 - 66.82 |  |
| Time since menopause |  |  |  |  | 0.294 |
| ≤20 y | 48.56 | 41.65 - 55.51 | 51.44 | 44.49 - 58.35 |  |
| >20 y | 55.04 | 43.59 - 66.11 | 44.96 | 33.89 - 56.41 |  |
| History of exogenous hormone use |  |  |  |  | 0.312 |
| Never | 44.11 | 31.88 - 56.89 | 55.89 | 43.11 - 68.12 |  |
| Ever | 50.42 | 44.09 - 56.74 | 49.58 | 43.26 - 55.91 |  |

Values are weighted row percentages with 95% CI. Column percentages for the sample totals that do not add up to 100% are a result of missing data. * *p*<0.05 vs. values in the group without breastfeeding history. PIR, prescribed investor rate; BMI, body mass index; y, years; CI, confidence interval.
